# Induced Resistance by a Long-Chain Bacterial Volatile: Elicitation of Plant Systemic Defense by a C13 Volatile Produced by *Paenibacillus polymyxa*

**DOI:** 10.1371/journal.pone.0048744

**Published:** 2012-11-28

**Authors:** Boyoung Lee, Mohamed A. Farag, Hyo Bee Park, Joseph W. Kloepper, Soo Hyun Lee, Choong-Min Ryu

**Affiliations:** 1 Molecular Phytobacteriology Laboratory, Systems and Synthetic Biology Research Center, KRIBB, Daejeon, South Korea; 2 Biosystems and Bioengineering Program, University of Science and Technology, Daejeon, South Korea; 3 Pharmacognosy Department, Faculty of Pharmacy, Cairo University, Cairo, Egypt; 4 Department of Entomology and Plant Pathology, Auburn University, Auburn, Alabama, United States of America; Virginia Tech, United States of America

## Abstract

**Background:**

Some strains of plant growth-promoting rhizobacteria (PGPR) elicit induced systemic resistance (ISR) by emission of volatile organic compounds (VOCs) including short chain alcohols, acetoin, and 2,3-butanediol. The objective of this study was to evaluate whether species-specific VOCs from PGPR strain *Paenibacillus polymyxa* E681 can promote growth and induce resistance in *Arabidopsis*.

**Methodology/Principal Findings:**

The efficacy of induction was strain-specific, with stronger protection against *Pseudomonas syringae* pv. maculicola ES4326 in plants exposed to VOCs from *P. polymyxa* E681 versus *Arabidopsis* plants exposed to VOCs from a reference strain *Bacillus subtilis* GB03, which was previously shown to elicit ISR and plant growth promotion. VOC emissions released from E681 primed transcriptional expression of the salicylic acid, jasmonic acid, and ethylene signaling marker genes *PR1*, *ChiB,* and *VSP2*, respectively. In addition, strain E681 produced more than thirty low molecular-weight VOCs, of which tridecane was only produced by E681 and not found in GB03 or IN937a volatile blends. These strain-specific VOCs induced *PR1* and *VSP2* genes.

**Conclusions/Significance:**

These results provide new insight into the existence of a long chain VOC signaling molecule produced by *P. polymyxa* that can serve as a bacterial trigger of induced systemic resistance *in planta*.

## Introduction

Plant growth-promoting rhizobacteria (PGPR) are a group of root-colonizing bacteria in the rhizosphere of many plant species that enhance plant productivity and often elicit plant immunity against multiple plant pathogens [1], [2], [3], a process referred to as induced systemic resistance (ISR). PGPR have been found to produce many bacterial determinants that promote plant growth and reduce disease [4], [5]. Reduced disease can be via antibiotics and other bacterial metabolites that directly impact pathogens [6], [7]. Bacterial cell surface compounds and secreted compounds that have been identified as bacterial determinants responsible for ISR [1], [2], [3], [8] are lipopolysaccharides, salicylic acid (SA), 2,4-diacetylphloroglucinol, and siderophores.

In 2003, volatile organic compounds (VOCs) emitted by two *Bacillus* spp. were shown to be novel determinants of both plant growth promotion and elicitation of ISR in *Arabidopsis* [7], [9], [10]. Ryu et al. (2003) demonstrated significant growth promotion of *Arabidopsis* by *Bacillus subtilis* strain GB03 and *B. amyloliquefaciens* strain IN937a [10]. Among VOCs released from strain GB03, 2,3-butanediol was found as a major component that promoted plant growth and elicited ISR against *Erwinia carotovora* subsp. *carotovora*. Several mutant lines of *Arabidopsis*, including brassinosteroid- and gibberellic acid-insensitive mutants, auxin-transport-deficient mutants, and cytokinin receptor-deficient mutants, were used to elucidate the signaling pathways that promote growth [10]. The VOCs tested did not promote plant growth in *cre1*, a cytokinin receptor-deficient mutant, suggesting that cytokinin signaling is essential for the promotion of plant growth in response to bacterial volatiles [10].

Further, a study using Affymetrix *Arabidopsis* AG GeneChip revealed that volatile emissions from strain GB03 differentially up-regulated more than 600 transcripts, which encoded proteins with various functions such as cell wall modification, primary and secondary metabolism, stress responses, and hormone regulation [11]. Exposure of *Arabidopsis* to VOCs from strains GB03 and IN937a also resulted in a significant reduction in disease severity caused by *E. carotovora* subsp. *carotovora via* an ethylene (ET)-dependent but salicylic acid -independent signaling pathway [9]. Using the *PR1* as an indicator gene for SA signaling, researchers found no role for SA signaling during ISR elicited by volatiles from strain GB03.

PGPR strain E681 was isolated from roots of winter barley (*Hordeum vulgare* L.) in southern Korea and identified as a potential biological control agent that could promote growth and elicit biological control activity in diverse crop systems. Seed treatment with E681 was found to suppress early seedling damping-off in cucumber (*Cucumis sativus* L.) and sesame (*Sesamum indicum* L.). It also increased biological control capacity in greenhouse for cucumber and the field for sesame [3], [12], [13]. From *in vitro* testing to field trials, strain E681 has been identified as an agent that can elicit biological control as well as increase grain yield [3]. One possible explanation for the promotion of growth by strain E681 is *via* production of plant growth regulators auxin and cytokinin [14], [15], [16]. Whole genome sequencing of strain E681 revealed that it encodes an entire set of genes related to the production of indole acetic acid (IAA) [17].

Previously, it was problematic to test hemibiotrophic pathogenic bacteria such as *P. syringae* pv. maculicola ES4326 on *Arabidopsis* grown under *in vitro* conditions considering that drop-inoculation cannot easily elicit typical symptoms. To determine the bacterial volatile-mediated ISR capacity of E681 against a semibiotrophic bacterial pathogen *P. syringae*, a method of dipping seedlings into a suspension of pathogenic bacteria was developed. A bioassay system using tightly closed I-plates was previously used to assess the effects of volatiles on plant growth and ISR [9], [10]. The overall objective of this study was to determine if species-specific VOCs from *P. polymyxa* strain E681 promote plant growth and induce resistance against *P. syringae* pv. maculicola ES4326 in *Arabidopsis*. Using hormonal mutant lines of *Arabidopsis*, we further elucidated a plant hormone signaling pathway responsible for growth promotion. Comparison of bacilli VOC profiles from strains GB03, IN937a, and E681 revealed that tridecane, a C13 hydrocarbon produced exclusively by strain E681 and not from other tested bacilli spp., was a bacterial determinant that played a novel role in meditating ISR by priming defense genes such as *PR1, VSP2*, and *ChiB*.

## Results

### Enhancement of *Arabidopsis* growth by *Paenibacillus polymyxa* E681 volatiles

**Figure 1:**
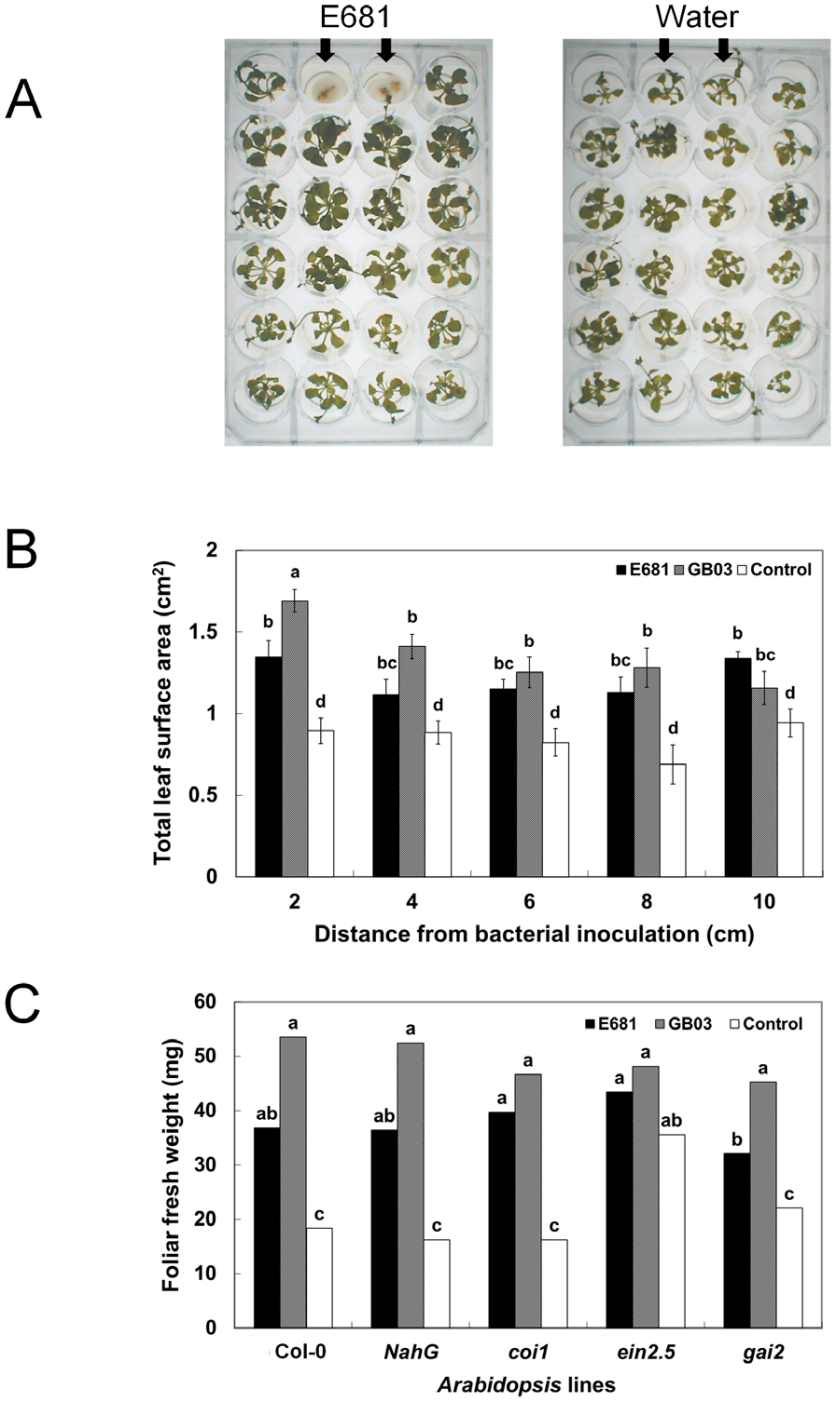
Growth promotion of *Arabidopsis* seedling by VOCs emitted from *Paenibacillus polymyxa* E681. A) Illustration of plant growth promotion by VOCs produced by strain E681 in a 24-well microtitre system. The diameter of each well was 2 cm. The photograph was taken two weeks after inoculation with strain E681. Right plate = water control; Left plate = E681 treatment. The single arrows indicate inoculation site with strain E681 and water control. B) Plant growth at two weeks after exposure to VOCs released by *P. polymyxa* E681, *Bacillus subtilis* GB03, and water control in a microtitre system, as indicated by the differences in the total leaf surface areas. C) Plant growth at two weeks after exposure to VOCs released by *P. polymyxa* E681, *Bacillus subtilis* GB03, and water control in a microtitre system, as indicated by the differences in the foliar fresh weight in wild type Col-0 and its hormonal mutant lines, transgenic NahG (encodes salicylate hydroxylase and degrades salicylic acid (SA)), *ein2.5* (ET-insensitive), *coi1* (insensitive to jasmonic acid), and *gai2* (insensitive to gibberellic acid). Different letters indicate significant differences between treatments at 2, 4, and 6 cm away from bacteria inoculation in the microtitre system, according to least significant difference at *P* = 0.05.

*P. polymyxa* E681 elicited the most growth promotion of *Arabidopsis* in the microtitre plate assay when the plant was located 2 cm from the bacteria (Figs. 1A and 1B). At this distance, total leaf surface area was increased 60% by strain E681 compared to control plants exposed to water. The total leaf surface area of *Arabidopsis* seedlings also increased following exposure to VOCs released from strains E681 and GB03 at all distances from the bacterial inoculation site. However, with strain GB03, growth of *Arabidopsis* seedlings placed 2 cm away from the bacteria increased more than seedlings grown at distances of 4, 6, 8, and 10 cm. This finding could be explained by the slow dispersal of the bacterial volatiles due to the lid being tightly closed. Another explanation could be that an insufficient concentration of VOCs delayed their ability to reach and affect plants grown further from the inoculation site.

To elucidate the mechanism underlying the observed plant growth promotion by bacterial VOCs, a series of mutant and transgenic *Arabidopsis* lines were assessed for responses to VOCs released by strains E681 and GB03. Results demonstrated differential plant growth promotion among various *Arabidopsis* lines. For example, plant growth was significantly enhanced by VOCs released from both bacterial strains in three *Arabidopsis* lines: coronatine/jasmonic acid (JA)-insensitive line, *coi1*, a SA-degrading line, NahG, and a gibberellic acid-insensitive line, *gai2* (Fig. 1C). In contrast, bacterial VOCs did not enhance growth of the ET- and cytokinin-insensitive *Arabidopsis* line, *ein2.5,* indicating growth promotion elicited by bacterial volatiles is mediated by the ET and or cytokinin signaling pathways (Fig. 1C).

### Elicitation of induced systemic resistance by bacterial volatiles in the microtitre system

**Figure 2:**
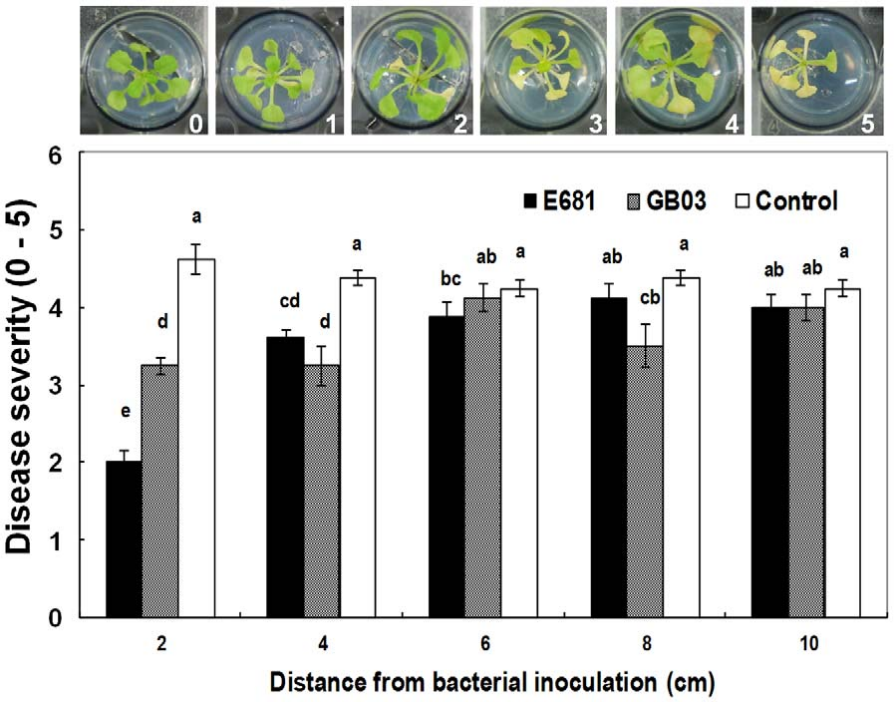
Induction of resistance against *Pseudomonas syringae* pv. maculicola ES4326 in *Arabidopsis* exposed to bacterial VOCs. Induced systemic resistance against *P. syringae* pv. maculicola ES4326 elicited by VOCs of *P. polymyxa* E681 and a water control, using the microtitre system. Disease severity (0 = no symptom, 10 = severe chlorosis) was recorded seven days after pathogen challenge. Different letters indicate significant differences between treatments, according to least significant difference at *P* = 0.05. The error bars indicate SEM.

As described in Materials and Methods, a new pathogenicity assay was developed, which was compatible with the microtitre plate system, using the foliar pathogen *Pseudomonas syringae* pv. maculicola ES4326 (*P. s. maculicola*). Soaking of *Arabidopsis* seedlings in a suspension of *P. s. maculicola* resulted in severe chlorosis at five days after inoculation and necrosis by seven days after inoculation (Fig. 2). The severity of disease was reduced two weeks after exposure of *Arabidopsis* seedlings to bacterial volatiles from strain E681 placed 2, 4, and 6 cm away from the seedlings in the microtitre system (Fig. 2). The maximum level of disease protection in this test occurred when *Arabidopsis* seedlings were placed 2 cm away from strain E681. VOCs released from PGPR strains E681 and GB03 also significantly reduced the disease severity from *P. s. maculicola* irrespective of distance from the PGPR inoculation site. The disease severity in *Arabidopsis* seedlings grown 10 cm away from E681 and GB03 did not differ relative to control treatment.

### Profiling of volatiles released by *Paenibacillus polymyxa* E681

**Figure 3:**
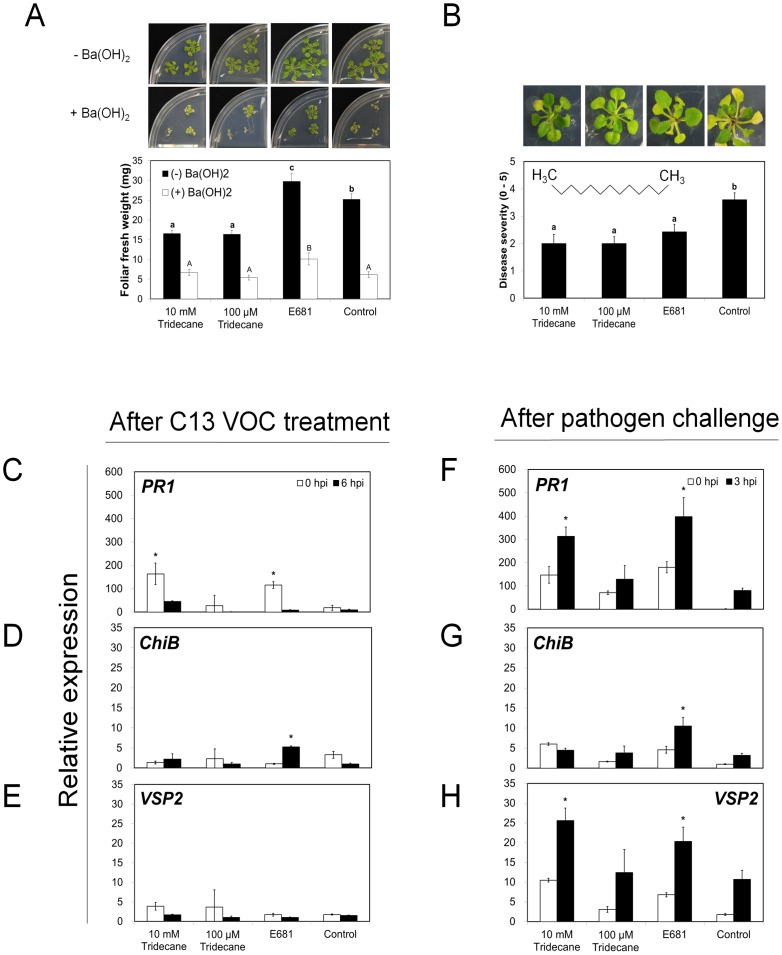
Induced systemic resistance and priming of defense-related genes by tridecane in *Arabidopsis*. A) Effect of tridecane on *Arabidopsis* growth. Plants were exposed to 10 mM and 100 µM tridecane, strain E681, and water for 2 weeks in presence (black bar) and absence of Ba(OH)_2_ (white bar). The photograph shows effect of CO_2_ on *Arabidopsis* growth treatments with 10 mM and 100 µM tridecane, strain E681, and water by the addition of Ba(OH)_2_ for trapping CO_2_ that precipitated as BaCO_3_. B) Induced systemic resistance against *P. syringae* pv. maculicola ES4326 elicited by strain E681 and 10 mM and 100 µM tridecane using the I-plate system. Disease severity (0 = no symptom, 5 = severe chlorosis) was recorded seven days after pathogen challenge at 10^8^ cfu/ml. Inset picture indicates chemical structure of tridecane. Gene expression levels of *PR1* for salicylic acid signaling (C and F), *ChiB* for ET signaling (D and G), and *VSP2* for jasmonic acid signaling (E and H), as determined by quantitative reverse transcriptase (qRT)-PCR after tridecane emission (C, D, and E) at 0 and 6 hour post-inoculation (hpi) and after pathogen challenge for detecting defense priming (F, G, and H). The expression ratio (C–H), a ratio of the expression in the strain E681 or tridecane-inoculated treatment relative to expression of *Actin* gene, is shown as the mean ± SEM. Different letters indicate significant differences between treatments (A and B) according to least significant difference at *P* = 0.05.

Solid-phase micro extraction (SPME) coupled with gas chromatography–mass spectrometry (GC–MS) was previously reported to be useful for profiling volatiles from PGPR strains [18]. Results from this analysis [18] provided a comprehensive compositional profile of VOCs released by strain IN937a and GB03. Therefore, it was used in the current study for profiling volatiles of strain E681. High levels of acetoin were consistently released from strains E681, IN937a, and GB03 (74, 153, and 226 µg/24 h, respectively), while the negative control strain 89B61 released only 0.1 µg/24 h (Table 1). Marginal detection levels of 2,3-butanediol were found in VOCs released by E681. Other volatiles released from the three bacilli strains GB03, IN937a, and E681 included methanethiol, isoprene, and acetic acid-butyl ester (Table 1). A comparison of the VOC profiles of strains GB03, IN937a, and E681 revealed that tridecane, a C13 hydrocarbon (MW = 184.35 daltons; Fig. 3B inset), was released exclusively from strain E681 (Table 1).

**Table 1:** Volatile profile of *Bacillus amyloliqefaciens* IN937a, *B. subtilis* GB03, and *Paenibacillus polymyxa* E681 using solid-phase micro extraction combined with gas chromatography–mass spectrometry.

RT (min)	Compounds^a^	VOC contents from PGPR strains (a.u. or µg)^b^ ^c^ ^d^		
		*B. amyloliquefaciens*	*B. subtilis*	*P. polymyxa*
Acohol				
4.49	Ethanol *$	10±3A	7±2A	2±0.3 BC
6.36	1-propanol-2-methyl *$	29±3 A	62±7A	6±1.5B
7.13	1-Butanol *$	3.6±0.6A	3.4±0.3A	0.5±0.04B
8.49	1-Pentanol *$	66±3A	0.4±0.04B	ND
8.56	1-Butanol-3-methyl *$	3961±214A	29±1B	19±1.5B
8.61	1-Butanol-2-methyl	290±28A	8±3B	0.9±0.07B
9.64	2,3-Butanediol *$	119±40A	257±138A	12±4B
Aldehydes				
6.87	Butanal-3-methyl	29±3A	77±5B	15±1C
7.04	Butanal-2-methyl *$	0.3±0.03A	0.06±0.01A	ND
12.37	2,4-Hexadienal	TR	TR	0.01±0.02
13.45	Benzaldehyde *$	0.003±0.04A	0.002±0.0003A	ND
Acids				
9.74	Glyoxylic acid	1±0.2 A	0.8±0.2A	0.7±0.1A
10.8	Acetic acid diethyl	6±0.5A	36±5B	2±0.1A
11.10	Butanoic acid-3-methyl *$	0.06±0.03A	0.3±0.1A	0.1±0.4B
Esters				
6.11	Ethyl acetate *$	21±3AB	49±10A	12±5A
10.12	Acetic acid butyl ester *$	2.8±0.2B	0.1±0.02A	4±1.5C
11.40	Butanol-3-methyl-acetate*$ 127	127±29	TR	TR
12.35	2-Butene-1-ol-3-methyl-acetate	0.15±0.01A	0.13±0.04A	0.1±0.02A
Ethers				
6.08	Furan-2-methyl *$	0.04±0.001A	0.1±0.009B	TR
7.50	Butane-1-methoxy-3-methyl *$	7530±512	ND	ND
7.60	Furan-2-ethyl *$	0.03±0.01	ND	ND
8.83	Furan-tetrahydro-2,5-dimethyl	0.2±0.02	TR	TR
13.70	Furan-2-pentyl *$	0.0005±0.0006A	0.0006±0.0006A	0.0003±0.0001A
Hydrocarbons				
4.86	Isoprene *$	483±109AB	614±62B	125±27A
7.53	Acetylene	9±0.8A	0.6±0.1B	0.12±0.01C
9.78	Cyclohexane	0.1±0.02A	0.2±0.07A	ND
15.42	1-Undecene	0.8±0.3A	1.2±0.1A	0.4±0.06AB
15.53	1-Undecane	1.5±0.12AB	3±0.2A	0.2±0.03BC
17.10	Dodecane	0.7±0.05A	0.9±0.07A	0.15±0.01B
18.52	Tridecane	ND	ND	0.14±0.01A
Ketones				
4.75	Acetone *$	0.2±0.03A	0.3±0.01A	0.5±0.5AB
5.74	2,3-Butanedione *$	38±4A	77±8A	14±3B
5.85	2-Butanone *$	24±4B	44±5A	12±2C
8.17	Acetoin *$	153±22A	226±37A	74±12A
10.16	2-hydroxy-3-pentanone *$	3.5±0.6A	3.5±0.7A	0.04±0.02B
S-containing compounds				
4.37	Methanethiol *$	0.6±0.1A	0.1±0.01A	0.3±0.09A
8.67	Dimethyl disulfide *$	4±0.1A	2.4±0.2B	2±0.2 BC
13.48	Dimethyl trisulfide	0.12±0.02	0.18±0.01A	0.1±0.01A
Inorganic compound				
4.03	Carbon dioxide	1912±40A	3907±150B	1277±246A

a Compounds marked with * were identified by comparison of retention time (RT) and mass spectral data with those of authentic compounds. The others were identified by comparison of mass spectral data with those of NIST library.

b Results are means of triplicate experiments; ND, not detected.

c Values for compounds marked with $ are concentrations, expressed in µg/24 h. Values unmarked are expressed as relative peak areas to (Z)-3-hexenyl acetate (IS) expressed in arbitrary units (a.u.). The volatile profiling of *B. amyloliquefaciens* and *B. subtilis* was adapted from the previous publication [18]. It should be noted that all volatile measurements were acquired at same time and under similar conditions from 3 PGPR.

d Values followed by the same letter within the same row are not significantly different (*P*>0.05).

It should be noted that enhanced growth promotion is unlikely to be mediated by CO_2_ emission levels (Table 1) from bacteria. Comparable CO_2_ emission levels were observed from *E. coli* DH5α (1277 µg/24 h) and active PGPR strain IN937a (1912 µg/24 h) [18]. Indeed, E681 released CO_2_ at emission levels almost similar to those released from the inactive *E. coli* DH5α [18]. Additional evidence that CO_2_ does not promote plant growth was observed from plants grown in the presence of Ba(OH)_2_, which captured released CO_2_ [19]. Additional evidence for a lack of a role of CO_2_ in growth promotion was found when the volatiles of strain E681 were found to still promote plant growth when exposed to Ba(OH)_2_ which captures CO_2_ (Fig. 3A). The difference in growth between with and without Ba(OH)_2_ treatments may be caused by elimination of total CO_2_ produced from tested plants and or from strain E681.

### Tridecane-elicited induction of systemic resistance against *P. syringae*

The application of tridecane at 10 mM and 100 µM elicited ISR and protected *Arabidopsis* seedlings against the biotrophic pathogenic bacterium *P. s. maculicola* at seven days after soaking-inoculation (Fig. 3). To determine if the dose of tridecane applied in this test was comparable to that released naturally from PGPR strain E681, quantification of tridecane levels in E681 headspace was attempted from small closed vials 20 mL (see Materials and Methods). The concentration of tridecane released from E681 in the 20 mL vial headspace reached 3.80 µM as determined using the SPME technique and tridecane standard doses analyzed under the same conditions (Table 1). The estimated concentration of introduced volatiles in this experiment reached *ca*.5.09 µM when the applied 30 µl of 10 mM tridecane was fully volatilized and had filled out the estimated volume of a Petri-dish (100 mm diameter×15 mm height). By comparison, the concentration of the introduced tridecane inside a Petri-dish was 1.34 times higher than that detected using SPME from strain E681 (Table 1). Therefore, it is highly like that tridecane application failed to inhibit bacterial growth, which would rule out a direct bacteriostatic effect (data not shown).

### Evaluation of tridecane on growth and priming plant defense gene expression

Volatile emissions from strain E681 promoted plant growth regardless of Ba(OH)_2_ treatment, whereas 10 mM and 100 µM tridecane decreased plant growth without Ba(OH)_2_ treatment and had no effect on plant growth with the presence of Ba(OH)_2_ compared to control (Fig. 3A). Interestingly, 10 mM dose of tridecane elicited plant protection at a level similar to that of 100 µM tridecane, suggesting that the ISR capacity against *P. s. maculicola* was not affected by difference in concentration levels (Fig. 3B). It is noteworthy that plants exposed to tridecane showed reduced fresh weight compared to the control, negating any role for tridecane in growth promotion.

To test for the induction of three defense genes, *PR1* for SA signaling, *VSP2* for JA signaling, and *ChiB* for ET signaling [20], qRT-PCR was conducted at 0 and 6 h after treatment with strain E681 and tridecane and at 0 and 3 h following challenge inoculation with bacterial pathogen *P. s. maculicola* challenge. Indirect (volatile) application of tridecane in I-plates did not significantly alter expression of *PR1*, *VSP2,* or *ChiB* (Figs. 3C, D and E). To evaluate defense priming of the 3 marker genes, gene expression levels were measured at 3 h following pathogen challenge. *Arabidopsis* was primed by *PR1, VSP2,* and *ChiB* gene expression through exposure to strain E681 volatile emissions, suggesting that SA, JA, and ET dependent signaling pathways were induced (Figs. 3F, G and H). Moreover, 10 mM tridecane resulted in a 4.7-fold increase in transcription of *PR1* compared to the water control before pathogen challenge (Fig. 3C) and 3.3-fold increase at 3 h after pathogen challenge (Fig. 3F). Similar results were observed for *PR1* gene in plants exposed to VOCs from strain E681 (Fig. 3F). At 10 mM, tridecane increased *VSP2* gene expression 2.5 fold compared to the control, albeit 100 µM tridecane showed no such effect on *PR1, VSP2,* and *ChiB* transcript levels (Figs. 3C to H). In summary, VOCs from PGPR strain E681 primed three defense genes - *PR1, VSP2,* and *ChiB* - which correspond to the elicitation of SA, JA, and ET signaling pathways. However, tridecane only induced *PR1* and *VSP2* after pathogen challenge. Tridecane at 10 and 100 µM did not lead to plant growth promotion but rather elicited ISR, suggesting that other unknown bacterial volatiles from strain E681 play a role in growth promotion.

### Induced resistance and defense priming by C10, C11, and C12 bacterial volatiles

**Figure 4:**
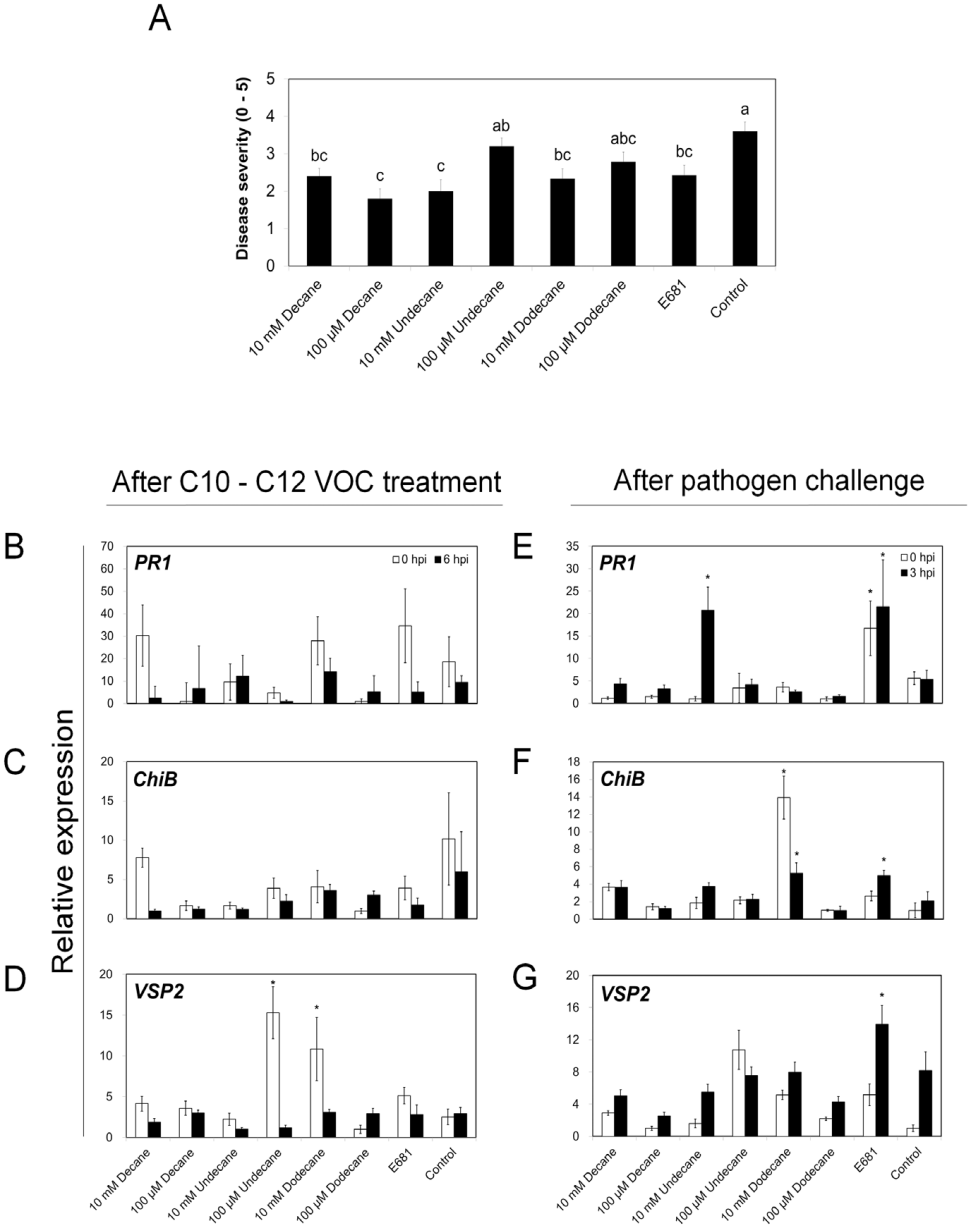
Induced resistance and priming of defense-related genes by long chain VOCs. A)Induced systemic resistance against *P. syringae* pv. maculicola ES4326 elicited by strain E681 and 10 mM and 100 µM of decane, undecane, and dodecane using the I-plate system. Disease severity (0 = no symptom, 5 = severe chlorosis) was recorded seven days after pathogen challenge at 10^8^ cfu/ml. Gene expression levels of *PR1* for salicylic acid signaling (B and E), *ChiB* for ET signaling (C and F), and *VSP2* for jasmonic acid signaling (D and G), as determined by quantitative reverse transcriptase (qRT)-PCR after tridecane emission (B, C, and D) at 0 and 6 hour post-inoculation (hpi) and after pathogen challenge for detecting defense priming (E, F, and G). The expression ratio (B–G), a ratio of the expression in the strain E681 or tridecane treatment relative to water-treated control, is shown as the mean ± SEM. Different letters indicate significant differences between treatments (A) according to least significant difference at *P* = 0.05.

The capacity of other long chain hydrocarbons that differ from tridecane by only one carbon unit, including C10, C11, and C12, to retain elicitation of plant defense against *P. s. maculicola*, was assessed. Accordingly, decane (C10), undecane (C11), and dodecane (C12), each at 10 mM and 100 µM, were tested in the I-plate system for elicitation of ISR against *P. s. maculicola.* All 3 hydrocarbons significantly decreased disease severity at 10 mM (Fig. 4A). The greatest ISR effects were observed with 100 µM decane and 10 mM undecane. These results indicate that some other long chain hydrocarbons can elicit ISR as does tridecane. Gene expression patterns mediating ISR were monitored in response to decane, undecane, and dodecane treatments in order to obtain further molecular evidence that the hydrocarbons mediated ISR. The expression patterns of *PR1*, *ChiB*, and *VSP2* expression pattern at 0 and 6 h post inoculation (hpi) were not significantly altered by any of the three hydrocarbons at 10 mM and 100 µM (Figs. 4B, C, and D). It should be noted that higher background expression levels of the three defense genes were detected at 0 h following application of 10 mM and 100 µM decane, undecane, and dodecane (Figs. 4B, C, and D). Only treatment with 10 mM undecane showed an increase, which was as high as 4.2- fold on mRNA transcriptional level of *PR1* gene compared to control at 3 hpi (Figs. 4E, F, and G).

## Discussion

The results reported here demonstrate for the first time that PGPR strain *Paenibacillus polymyxa* E681 produces a volatile blend that can enhance plant growth and elicit ISR against *P. syringae* in the absence of physical contact with plants (Fig. 1B). A new VOC assay system, based on a 24-well microtitre plate, was developed for studying the effects of volatiles produced by bacteria *in planta* (Figs. 1A, B and C). To overcome the difficulty of testing biotrophic bacteria such as *P. syringae*, a new seedling-dip method was developed that resulted in consistent symptom development of bacterial speck caused by *P. syringae*. Using this test system, we found that ISR elicited by VOCs released from strain E681 (Fig. 2) is mediated by priming of the defense genes *PR1*, *ChiB*, and *VSP2* (Figs. 3B to H).

To elucidate signaling pathways involved in growth promotion in response to VOCs, we evaluated *Arabidopsis* mutant lines that were defective in responding to known volatile signal molecules such as jasmonic acid (methyl jasmonate), ET, and salicylic acid (methyl salicylate). We cannot exclude the possibility that plant growth promotion by bacterial volatiles was in part due to elevated CO_2_ levels in the closed Petri dish system [19]. However, additional experiments revealed that strain E681 still enhanced plant growth when CO_2_ was captured with Ba(OH)_2_, a finding suggesting that CO_2_ may not be the only factor accounting for growth promotion(Fig. 3B).

An unexpected finding was that the ISR response against *P. syringae* pv. maculicola ES4326 was stronger in plants exposed to VOCs emitted from strain E681 than from strain GB03. In tobacco, 2,3-butanediol elicited ISR against the necrotrophic bacterium *Erwinia carotovora* subsp. *carotovora* but not against the biotrophic bacterial pathogen *P. syringae* [21]. This result led us to investigate whether the unique VOC blend released from strain E681 can more effectively elicit ISR response against pathovars of *P. syringae via* the elicitation of SA signaling pathway. To obtain a more detailed understanding of the underlying mechanism, we previously established that VOCs produced by strain GB03 triggered ET-dependent signaling in the exposed plant [9]. Interestingly, VOCs released by strain E681 significantly (*P* = 0.05) increased GUS activity of the promoter of *PR1*, an indicator of systemic acquired resistance or SA signaling (Figure S1).

To our knowledge, the only other bacterial species known to produce tridecane is *Pseudomonas aeruginosa* [22]. Aside from its production in bacteria, tridecane was also identified as an insect pheromone [23] and defensive signaling molecule by stink bug [24], [25], [26]. To date, microbial production of this hydrocarbon has not been extensively studied, and a plant's response to tridecane has not been assessed. How plants perceive and respond to tridecane has yet to be fully elucidated using large scale gene expression techniques by screening *Arabidopsis* mutants and transcriptional/proteomic changes by tridecane treatment. It remains to be determined whether tridecane is released in the rhizosphere where PGPR normally reside and whether ISR elicited by VOCs from strain E681 occurs in soil or soil-less media.

Production of cytokinin by *P. polymyxa*, or by plants exposed to *P. polymyxa*, was previously proposed as a bacterial determinant in growth promotion [16]. Our data presented here indicate that bacterial volatiles emitted from *P. polymyxa* E681 play an important role in growth promotion of *Arabidopsis* seedlings. Moreover, out of a blend of 30 VOCs, tridecane was found to elicit ISR against *P. syringae* pv. maculicola strain ES4326. Interestingly, tridecane has a negative effect on plant growth promotion (Fig. 3), indicating that different VOCs from bacteria play a role in plant growth promotion and ISR in plants. The qRT-PCR results indicated that tridecane emitted from strain E681 may elicit a plant defense mechanism *via* SA and or JA signaling pathways (Figs. 3F, G, and H). For unequivocal proof of the involvement of respective defense signaling pathways, other signaling mutant lines of *Arabidopsis* need to be assessed in response to VOCs emission. Further studies are needed to evaluate results between decreases in disease severity following exposure to other VOCs like decane and undecane and priming of candidate defense genes (Figs. 4E, F, and G).

Plants may perceive diverse VOCs with similar structures at a given threshold level and prime diverse unknown downstream signaling pathways related to plant defense. In addition to ISR response, bacterial volatiles emitted from strain GB03 were recently reported to elicit an abiotic stress tolerance to salt stress, depending on activity of high-affinity K^+^ transporter 1 (HKT1), which manipulates Na^++^ homeostasis in the plant root system [27]. Similarly, application of *Pseudomonas chlororaphis* O6 to the root elicited ISR to biotic and abiotic stresses *via* volatile emission. Intriguingly, 2,3-butanediol, produced by *P. chlororaphis*, mediated stomata closure and drought resistance in *Arabidopsis* in an Aba-1- and OST-1 kinase-dependent manner [28]. It is noteworthy that certain concentrations of a long chain hydrocarbon provide ISR against *P. syringae*, which suggests that the hydrocarbon is utilized as a biochemical trigger of induced systemic tolerance against abiotic stresses as well as induced resistance for managing plant diseases [29]. Finally, results presented herein provide evidence that strain E681 emits a long chain C13 volatile that can trigger a stronger ISR response than 2,3-butanediol or acetoin, and suggesting for the presence of other unidentified molecules that have yet to be identified and examined in large-scale field experiments.

### Conclusion

Overall, the results provide new evidence that a C13 hydrocarbon, tridecane, emitted from *Paenibacillus polymyxa*, can promote plant growth *via* ET-dependent signaling pathway and can induce systemic resistance by priming of plant defense-related genes. In addition, this study provides new insight into the role of a species-specific long-chain bacterial volatile as a trigger of both growth promotion and defense *in planta*. The observed variation in volatile profiles among various PGPR strains suggests that a diverse VOC metabolism exists among PGPR and supports the idea that VOCs can serve as taxonomic markers in microbial systems [30]. Coupling these differential VOC profiles from different PGPR strains with the levels of gene transcription could prove useful for probing biosynthetic pathways leading to tridecane production in *P. polymyxa*.

## Materials and Methods

### Bacteria and plant preparation

PGPR strains *Pseudomonas fluorescens* 89B-61, *B. amyloliqefaciens* IN937a, *Bacillus subtilis* GB03, and *Paenibacillus polymyxa* E681 were streaked onto tryptic soy agar (TSA, Difco Laboratories, Detroit, MI, USA) plates and incubated for 24 hours in darkness at 28°C. Strain E681 was previously isolated from the roots of winter barley in the southern part of Korea. For long-term storage, bacterial cultures were maintained at −80°C in tryptic soy broth (TSB) (Difco Laboratories, Detroit, MI, USA) that contained 20% glycerol. *Arabidopsis thaliana* ecotype Columbia (Col-0) plants were prepared as described previously [2], [9], [10]. Foliar fresh weight was used as a measure of the growth parameter for plant growth promotion in *A. thaliana* ecotype Col-0. All chemicals including decane, undecane, dodecane, and tridecane were purchased from Sigma Aldrich.

### Assessment of plant growth promotion by bacterial volatiles

In a preliminary experiment, the capacity of strains GB03, 89B61, and E681 to promote growth of wild type and mutant *Arabidopsis* plants was assessed using the previously described I-plate system [9], [10]. To test the effect of bacterial volatiles on growth and ISR at different distances from the bacterial inoculation site, a 24-well microtitre plate system was developed. An *Arabidopsis* seedling was placed into each well, which contained 1 ml of half-strength MS medium. The two wells of the first row were then inoculated with a bacterial suspension at 10^8–9^ colony forming units (cfu) per ml as shown in Fig. 1A to avoid physical contact between bacterial inoculation and plant. To assess the capacity of bacterial VOCs to promote plant growth, the total leaf surface area (cm^2^) of each seedling growing 2, 4, 6, 8, and 10 cm from the bacterial inoculation site was measured two weeks after bacteria inoculation.

To exclude CO_2_ effect, 5 mL of 0.3 N Ba(OH)_2_ was applied on one part of a 4-part divided Petri dish [19]. At 2-weeks after cultivation of plants in the presence of 0.3 N Ba(OH)_2_, fresh weights of each aerial part of plants were measured. The mean value of plant fresh weight was compared with the mean fresh weight of plants cultivated without Ba(OH)_2_ (Fig. 3A).

### Assessment of induced systemic resistance by bacterial volatiles

To determine if exposure of *Arabidopsis* seedlings to bacterial volatiles elicited ISR against *Pseudomonas syringae* pv. maculicola ES4326, we developed a 24-well microtitre-based disease assay system (Fig. 1A). The 24-multimicrotitre plate of bacterial suspensions of PGPR strains *B. subtilis* GB03 and *P. polymyxa* E681 at OD_600_ = 1 (10^8–9^ cfu/ml) was inoculated in an empty well (without plant). For assessing ISR by bacterial volatile and its derivatives, the I-plate system was employed. After application of 30 µl of 10 mM and 100 µM decane, undecane, and dodecane, the I-plate was tightly sealed with parafilm. Seven days after inoculation with PGPR or VOCs, 2.5 ml of a bacterial suspension (at OD_600_ = 1) of *P. syringae* pv. maculicola ES4326 grown on King's B medium was added to each well. A whole seedling in each well was then soaked in the bacterial suspension for 5 min, and the suspension was removed. The plant was rinsed with sterile distilled water three times. The 24-multimicroliter plate with challenged *Arabidopsis* seedlings was then placed in a growth chamber that was maintained at 21°C in 12/12 day and night condition. Disease severity was measured four to seven days after pathogen challenge.

### Analysis of bacterial volatiles

*Bacillus subtilis* strain GB03, *B. amyloliqefaciens* IN937a, *P. polymyxa* strain E681, and *P. fluorescens* strain 89B61 were grown in 20 ml vials on MS medium containing 1.5% (w/v) agar, 1.5% (w/v) sucrose, and 0.4% (w/v) TSA for 24 h at 37°C before volatiles were collected. Vials were sealed with a steel crimp cap fitted with a Teflon/silicon septum that was previously conditioned at 100°C for 30 minutes. (Z)-3-Hexenyl acetate, which is absent from bacterial cultures, was used as an internal standard (IS) and was injected at a concentration of 1 µg per vial. Solid-phase microextraction (SPME) and gas chromatography–mass spectrometry (GC-MS) analysis were performed as previously described [18]. Briefly, vials containing samples were placed in a heating block (Gerstel Multi Purpose Sampler MPS 2, Baltimore, MD) at 50°C with a SPME fiber (stable flexdivinylbenzene/carboxen/PDMS (DCP, 50/30 µm)) inserted into the headspace above the bacterial sample. Adsorption was timed for 30 minutes, and fibers were desorbed at 210°C for 1 min in the injection port of an HP 5890A GC/MS (Hewlett-Packard, Palo Alto, CA) with a DB-5 column (60 m, 0.25 mm i.d., 0.25 µm film thickness) (J&W Scientific, Folsom, CA). The HP quadrupole mass spectrometer was operated in the electron ionization mode at 70 eV, a source temperature of 200°C, quadrupole temperature of 150°C, with a continuous scan from m/z 40 to 500. Positive identification of each chemical constituent was performed by comparison of its retention time and mass spectrum with that of authentic standard (when available). Tentatively identified compounds were uniquely identified on the basis of EPA/NIH database. Peaks were quantified by selected abundant fragments (m/z) to overcome the problem of co-eluted compounds [18]. Samples were run in triplicate, and integrated areas were normalized on (Z)-3-hexenyl acetate and averaged. Fourteen compounds were quantified using calibration curves of authentic standards. Quantification of volatiles' components was carried out with response factors (RFs). RFs and calibration curves were determined by diluting standards in water at four concentrations (0.1, 1, 10, and 100 µg/ml) as described by Farag et al. [18].

### Quantitative Reverse Transcriptase (qRT)-polymer chain reaction

Total RNA was isolated from inoculated 14-day-old leaf tissues according to the protocol of Yang et al [29]. The purified total RNA was treated with 1 U of RNase-free DNase (Promega, USA) for 10 minutes (min) at 37°C and then subjected to a second round of purification using the TRI reagent. First-strand cDNA synthesis was carried out with 2 µg of DNase-treated total RNA, oligo dT primers, and Moloney murine leukemia virus reverse transcriptase (MMLV- RT; Invitrogen, USA). Expression of the candidate gene was analyzed using the following primers: 5′-TTCCACAACCAGGCACGAGGAG-3′ (*PR1*_F), 5′-CCAGACAAGTCACCGCTACCC-3′ (*PR1*_R); 5′-CCCTAAAGAACGACACCGTCAA-3′ (*VSP2*_F), 5′-TCAATCCCGAGCTCTATGATGTTT-3′ (*VSP2*_R). AGI codes were *PR1* (AT2G14610), *VSP2* (AT5G24770), *CHIB* (AT3G12500), and *AtACT2* (AT3G18780) as a control to ensure that equal amounts of RNA were analyzed in each experiment. *AtActin* was also assayed using the primers 5′-GTTAGCAACTGGGATGATATGG- and 5′-CAGCACCAATCGTGATGACTTGCCC-3′. Candidate priming genes were amplified from 100 ng of cDNA by PCR using an annealing temperature of 60°C. Q-RT-PCR was carried out using a Chromo4 real-time PCR system (Bio-Rad). Reaction mixtures (10 µl) contained 5 µl of 2×Brilliant SYBR Green QPCR master mix (Bio-Rad), cDNA, and 1 pmole of each primer. The thermocycle parameters were as follows: initial polymerase activation for 10 min at 95°C, and then 40 cycles of 30 seconds (s) at 95°C, 30 s at 60°C, and 30 s at 72°C. Conditions were determined by comparing the threshold values in a series of dilutions of the RT product, followed by a non-RT template control and a non-template control for each primer pair. Relative RNA levels were calibrated and normalized to the level of *AtAct2* mRNA.

### Statistical Analysis

Analysis of variance for experimental data sets was performed using JMP software version 4.0 (SAS Institute Inc., Cary, NC). Significant treatment effects were determined based on the magnitude of the *F* value (*P* = 0.05). When a significant *F* test was obtained for treatments, separation of means was accomplished by Fisher's protected least significant difference (LSD) at *P* = 0.05. Bioassays were conducted at least twice, with eight replicates per treatment and one seedling per replicate. For VOC analyses, four replicates per bacterial culture measured were performed.

## Supporting Information

Figure S1 **GUS activity in** ***Arabidopsis*** **plants transformed with** ***ProPR-1::GUS*** **or** ***PDF1.2::GUS*** **that were exposed to VOCs released from** ***P. polymyxa*** **E681 and** ***B. subtilsis*** **GB03.** Induction of *PR1* and *PDF1.2* promoters fused with GUS by *P. polymyxa* E681 and *B. subtilis* GB03. Different letters (a, b for *PR-1a*; x, y for *PDF1.2*) indicate significant differences between treatments within each *Arabidopsis* line, according to least significant difference at *P* = 0.05. The error bars indicate S.E.M.
(TIF)
