# Supplementary figures and images for: Induced Resistance by a Long-Chain Bacterial Volatile: Elicitation of Plant Systemic Defense by a C13 Volatile Produced by Paenibacillus polymyxa
Source: PLoS One. 2012 Nov 28;7(11):e48744. doi: 10.1371/journal.pone.0048744 (PMC3509098; doi:10.1371/journal.pone.0048744)

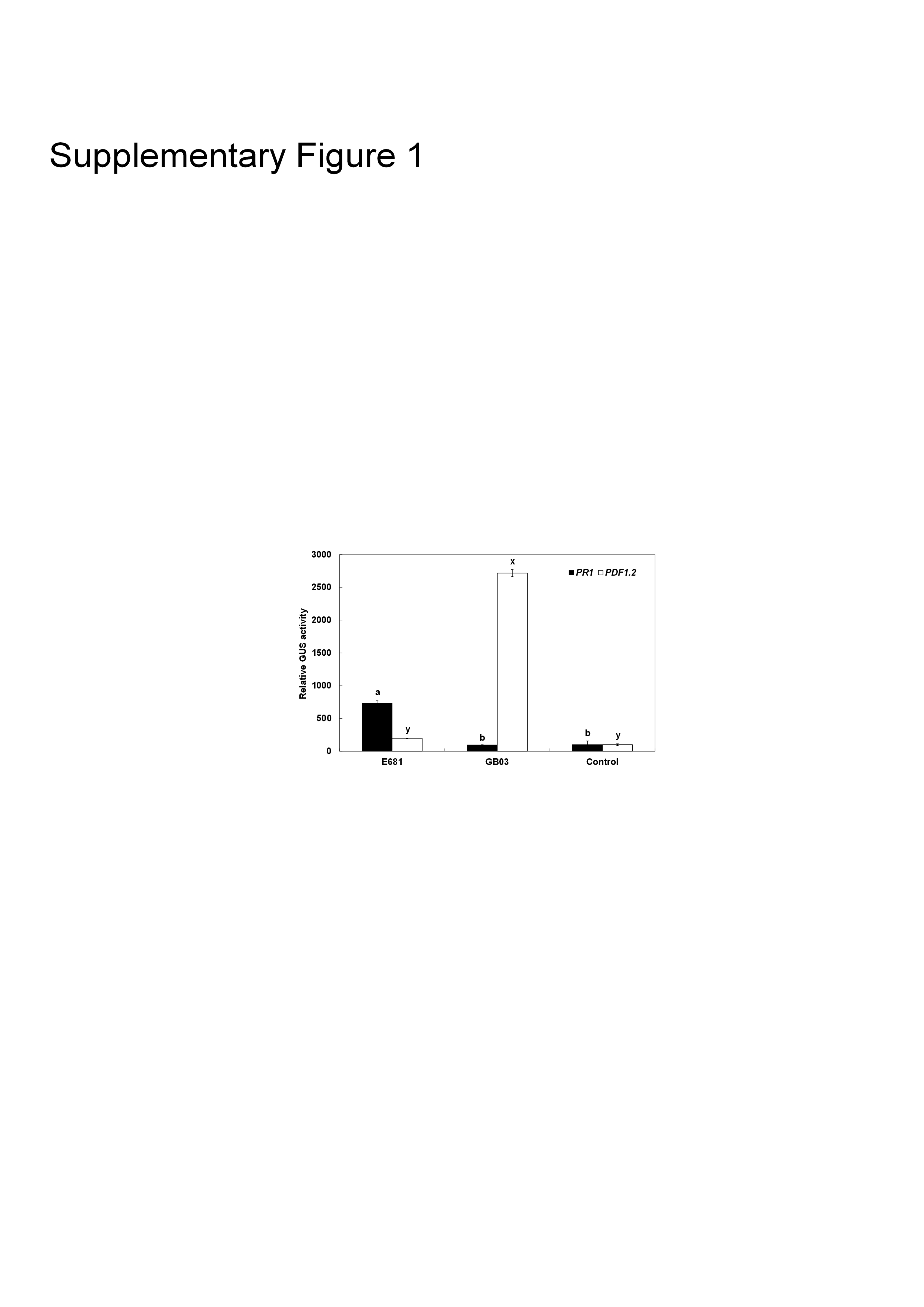

Supplement: Figure S1 — GUS activity in Arabidopsis plants transformed with ProPR-1::GUS or PDF1.2::GUS that were exposed to VOCs released from P. polymyxa E681 and B. subtilsis GB03. Induction of PR1 and PDF1.2 promoters fused with GUS by P. polymyxa E681 and B. subtilis GB03. Different letters (a, b for PR-1a; x, y for PDF1.2) indicate significant differences between treatments within each Arabidopsis line, according to least significant difference at P = 0.05. The error bars indicate S.E.M. (TIF) [file pone.0048744.s001.tif]
